# Intraventricular Hemorrhage and Early Hematoma Expansion in Patients with Intracerebral Hemorrhage

**DOI:** 10.1038/srep11357

**Published:** 2015-06-18

**Authors:** Qi Li, Yuan-Jun Huang, Gang Zhang, Fa-Jin Lv, Xiao Wei, Mei-Xue Dong, Jian-Jun Chen, Li-Juan Zhang, Xin-Yue Qin, Peng Xie

**Affiliations:** 1 Department of Neurology, The First Affiliated Hospital of Chongqing Medical University, Chongqing 400016, China; 2 Department of Radiology, The First Affiliated Hospital of Chongqing Medical University, Chongqing 400016, China; 3 Department of Medical Technology, Chongqing Medical and Pharmaceutical College 400030, Chongqing, China

## Abstract

Intraventricular hemorrhage is associated with poor functional outcomes in patients with intracerebral hemorrhage (ICH). We aimed to investigate the association between intraventricular hemorrhage and early hematoma expansion in patients with ICH. Patients with ICH who underwent a baseline CT scan within six hours after onset of symptoms were included. The follow-up CT scan was performed within 24 hours after the baseline CT scan. Univariate and multivariable logistic regression were used to assess the relationship between presence of intraventricular hemorrhage and early hematoma expansion. A total of 160 patients were included in the study. Significant hematoma growth was observed in 52 (32.5%) patients presenting within six hours after onset of symptoms. Intraventricular hemorrhage was observed in 66 (41.25%) patients with ICH. Multivariate analyses demonstrated that a short time from onset to baseline CT scan, the initial hematoma volume, and the presence of intraventricular hemorrhage on follow-up CT scan were independently associated with hematoma enlargement. The presence of intraventricular hemorrhage on follow-up CT scan can be associated with hematoma expansion in patients with ICH.

Hematoma expansion, which is defined as an increase in hematoma volume of >33% or an absolute increase of hematoma volume of >12.5 mL, is a relatively common complication after ICH^1^. Early hematoma expansion has been observed in greater than one-third of patients within the first few hours of intracerebral hemorrhage^2^,^3^,^4^,^5^,^6^,^7^. It is well-documented that early hematoma expansion is independently associated with neurological deterioration and poor functional outcome^8^.

Previous studies have suggested that intraventricular hemorrhage is not uncommon in patients with intracerebral hemorrhage and is associated with poor functional outcomes^9^,^10^. However, few studies have investigated the association between intraventricular hemorrhage and hematoma expansion^11^,^12^. In a study of 627 patients, Fujii *et al.* reported that intraventricular hemorrhage does not predict hematoma expansion in patients with spontaneous ICH^11^. In another study of 183 patients, Silva and colleagues observed that intraventricular bleeding is associated with early ICH growth^12^. Thus, it is important to investigate the association between hematoma expansion and intraventricular hemorrhage, as this research may provide new targets for therapeutic intervention. Therefore, the aim of the study was to investigate the association between intraventricular hemorrhage and early hematoma expansion in patients with intracerebral hemorrhage.

## Results

One hundred and sixty patients (110 males, 50 females) met the inclusion criteria and were included in our final analysis. Their mean age was 60.3 ± 12.4 years (age range: 27–87 years). The median baseline ICH volume was 12.7 ± 15.2 mL. The median follow-up ICH volume was 16.1 ± 37.4 mL. The median time from symptom onset to baseline CT scan was 120 ± 99 minutes. The median time from baseline CT scan to follow-up CT scan was 17.5 ± 7.6 hours. The baseline hematoma location was deep in 132 (82.5%) patients, lobar in 20 (12.5%) patients, brainstem in 4 (2.5%) patients, and cerebellar in 4 (2.5%) patients. The location of the hematoma was supratentorial in 152 patients and infratentorial in 8 patients.

The demographic, clinical, and radiological characteristics of patients with and without hematoma expansion are compared and listed in Table 1. The age, gender, history of hypertension, diabetes, smoking history, and alcohol consumption were similar between patients with hematoma expansion and those without expansion. The baseline hematoma volume was significantly larger in patients with hematoma expansion (P < 0.01). Patients with early hematoma expansion had a shorter time to initial CT scans as compared with those without hematoma expansion (P < 0.01).

Intraventricular hemorrhage was observed in 66 (41.25%) patients with ICH. Of the 66 patients, 57 (86.3%) had intraventricular hemorrhage on baseline CT scan. Nine patients (13.7%) had delayed intraventricular hemorrhage. Significant hematoma growth was observed in 52 (32.5%) patients presented within 6 hours after onset of symptoms. Of the 52 patients, 30 (57.7%) had intraventricular hemorrhage. Intraventricular hemorrhage was observed in 36 (33.3%) of 108 patients without hematoma expansion. Intraventricular hemorrhage was more common in patients with hematoma expansion than those without hematoma expansion on follow-up CT scan (P = 0.003).

The results of the univariate and multivariate logistic regression analysis are listed in Table 2 and Table 3. In the univariate logistic analysis, the time to baseline CT scan (OR 0.62; 95% CI: 0.48–0.81), initial hematoma volume (OR 1.07; 95% CI: 1.04–1.1), and the presence of intraventricular hemorrhage on follow-up CT scan (OR 2.72; 95% CI: 1.38–5.38) were associated with early hematoma expansion. The multivariate regression analysis confirmed that the time to baseline CT scan (OR 0.59; 95%CI: 0.46–0.79), initial hematoma volume (OR 1.07; 95%CI: 1.04–1.12) and presence of intraventricular hemorrhage on follow-up CT scan (OR 2.35; 95%CI: 1.17–5.17) were associated with early hematoma expansion.

## Discussion

In our study, we have identified several parameters that are associated with early hematoma expansion. Consistent with previous reports^5^,^6^, we found that the initial baseline hematoma volume is an independent predictor for early hematoma expansion. The presence of a large hematoma at baseline CT scan may increase the effect of vessel shearing, leading to hematoma expansion^13^. We also found that a short time to baseline CT scan is an independent predictor for early hematoma expansion. Since most hematoma expansion occurs within the first hours after onset of symptoms, patients who receive early CT imaging are more likely to have hematoma expansion^14^.

To date, several predictors for hematoma expansion have been identified. Large hematoma volume, short time to baseline CT scan, and a CT angiography spot sign are well-established imaging predictors for hematoma expansion^8^,^9^,^10^,^11^,^12^. That being said, the association between blood pressure and early hematoma expansion remains controversial. Some investigators report a possible association between elevated blood pressure and hematoma enlargement^4^,^15^. However, the association has not been established in other studies^16^,^17^. In line with these latter studies, we found that neither history of hypertension nor blood pressure are associated with early hematoma expansion.

In this study, we investigated the relationship between intraventricular hemorrhage and early hematoma expansion. We found an association between the presence of intraventricular hemorrhage on a follow-up CT scan and early hematoma expansion. However, an association between intraventricular extension of ICH on initial CT scan and early hematoma expansion was not established. Intraventricular hemorrhage is not uncommon in patients with ICH. It is reported in approximately 30%–50% of patients with ICH^18^,^19^. Furthermore, the intraventricular extension of hemorrhage is an important independent predictor of poor outcome in patients with ICH^11^,^12^.

Recent research suggests that delayed intraventricular hemorrhage is associated with poor functional outcomes in patients with ICH^20^. Early intraventricular hemorrhage, which is defined as presence of ventricular blood on an initial CT scan, has been observed in 21 patients with hematoma expansion and does not predict hematoma expansion. In our study, we found delayed intraventricular hemorrhage is observed in 9 patients with hematoma expansion. Furthermore, all patients with delayed intraventricular hemorrhage in our cohort had hematoma expansion. This phenomenon may partially explain why delayed intraventricular hemorrhage is associated with poor functional outcomes in patients with ICH.

Our study has several limitations. Firstly, the admission-based Glasgow Coma Scale, which has been shown to be predictive of 30-day mortality, was not included in our analysis. Secondly, the association between ICH and functional outcome was not analyzed here. Thirdly, the sample size is relatively small. Future studies with large sample size and functional follow-up are needed to further clarify the relationship between intraventricular hemorrhage and hematoma expansion in patients with ICH.

In conclusion, this study finds an association between intraventricular hemorrhage on follow-up CT scan and early hematoma expansion. Our findings suggest that the presence of intraventricular hemorrhage on CT scan may indicate prior significant hematoma expansion.

## Methods

### Patients

This study was approved by the Ethics Committee of Chongqing Medical University. Informed consent was obtained from all participants or their close relatives. The study protocol was performed in accordance with relevant ethical guidelines and regulations for human studies. Only patients aged older than 18 years who presented with acute ICH between June 2011 and September 2014 to our institution were included in our study. Patients were eligible if (i) they underwent baseline head CT scan within six hours after onset of symptoms and (ii) follow-up CT scans were performed within 24 hours after the baseline CT scan.

Patients were excluded from the study if: (i) the ICH was secondary to brain tumor or hemorrhagic transformation of a cerebral infarction, (ii) they displayed wake-up stroke, (iii) they presented after six hours post-onset of symptoms, (iv) they presented with isolated intraventricular hemorrhage, (v) they recently received anticoagulants or antithrombotic therapy before onset of ICH, or (vi) they had neurosurgical treatment before follow-up CT scan.

The baseline demographics, past medical history, and vascular risk factors of the recruited patients were recorded. The time to initial CT scan was also recorded for each participant. Blood pressure was monitored and recorded immediately after admission.

### Imaging Analysis

Baseline and 24-hour follow-up CT scans were performed using a multi-detector CT scanner. CT scans were performed on a 512 × 512 matrix with a 5-mm section thickness. Licensed neurologists who were blinded to the clinical data identified ICHs and intraventricular hemorrhage on the CT scans. The hemorrhage locations were assessed and categorized as “deep”, “lobar”, “brainstem”, or “cerebellar” hemorrhages. “Deep” intracerebral hemorrhages were defined as hemorrhages involving the basal ganglia, thalamus, internal capsule, or deep white matter. The baseline and follow-up CT scans were used to calculate the hematoma volume. Significant hematoma expansion was defined as an increase of hematoma of greater than 33% or an absolute increase of greater than 12.5 mL from the baseline CT scan.

### Statistical Analysis

The demographic, clinical, and radiological characteristics were compared between expanders and non-expanders using Chi-square and Student’s t-testing as appropriate. All statistical tests were two-tailed with a P-value of less than 0.05 considered statistically significant. All statistical analyses were performed using SPSS version 19.0. Univariate and multivariate logistic regression models were used to assess the association of intraventricular hemorrhage with significant hematoma expansion. Variables with statistical significance in the univariate analysis and potential confounders were included in the multivariate logistic regression model for analyzing the association between intraventricular hemorrhage and hematoma expansion.

## Additional Information

**How to cite this article**: Li, Q. *et al.* Intraventricular Hemorrhage and Early Hematoma Expansion in Patients with Intracerebral Hemorrhage. *Sci. Rep.* **5**, 11357; doi: 10.1038/srep11357 (2015).

## Figures and Tables

**Table 1:** Comparison of Baseline Demographic, Clinical, and Radiological Characteristics between Patients with and without Hematoma Expansion.

Characteristics	Expander (n = 52)	Non-expander (n = 108)	P-value
Mean age, years (SD)	62.7 (11.7)	59.1 (12.5)	0.108
Current smoker (%)	25 (48.1)	48 (44.4)	0.665
Alcohol consumption (%)	22 (42.3)	50 (46.3)	0.635
Gender, Male (%)	39 (75)	71 (65.7)	0.237
Hypertension (%)	36 (69.2)	68 (62.9)	0.436
Diabetes mellitus (%)	8 (15.3)	14 (12.9)	0.677
Supratentorial hematoma (%)	51 (98.1)	101 (93.5)	0.218
Time to baseline CT (SD)	1.72 (1.20)	2.77 (1.73)	<0.001
Baseline ICH volume (SD)	25.65 (21.49)	12.72 (8.19)	<0.001
IVH on baseline CT (%)	21 (40.3)	36 (33.3)	0.386
IVH on follow-up CT (%)	30 (57.6)	36 (33.3)	0.003

**Table 2:** Univariate Logistic Regression Analysis of Intraventricular ICH and Hematoma Expansion.

Variable	Odds Ratio	95% CI	P-value
Age	1.02	0.99 – 1.05	0.08
Gender	0.64	0.31 – 1.34	0.23
Current smoking	0.86	0.45 – 1.67	0.66
Alcohol consumption	1.17	0.60 – 2.29	0.63
Hypertension	0.75	0.37 – 1.53	0.43
Systolic Blood Pressure	1.01	0.99 – 1.02	0.19
Diastolic Blood Pressure	1.01	0.98 – 1.03	0.69
Diabetes mellitus	0.81	0.32 – 2.09	0.67
Supratentorial hematoma	0.28	0.03 – 2.36	0.24
Time to baseline CT	0.62	0.48 – 0.81	<0.001
Baseline ICH volume	1.07	1.04 – 1.1	<0.001
IVH on baseline CT scan	0.73	0.37 – 1.46	0.38
IVH on follow-up CT scan	2.72	1.38 – 5.38	0.004

**Table 3:** Multivariate Logistic Regression Analysis of Intraventricular ICH and Hematoma Expansion.

Variable	Odds Ratio	95% CI	P-value
Time to baseline CT	0.59	0.46 – 0.79	0.001
Baseline ICH volume	1.07	1.04 – 1.12	<0.001
IVH on follow-up CT scan	2.35	1.17 – 5.17	0.03
